# Fuzzy Logic System Assisted Sensing Resource Allocation for Optical Fiber Sensing and Communication Integrated Network

**DOI:** 10.3390/s22207708

**Published:** 2022-10-11

**Authors:** Chenlin Zhang, Pan Wang

**Affiliations:** 1 School of Computer Science, Chengdu University of Information Technology, Chengdu 610225, China; 2 School of Information and Communication Engineering, University of Electronic Science and Technology of China (UESTC), Chengdu 611731, China

**Keywords:** optical fiber sensing, fiber-optic communication network, fuzzy logic system, sensing resources allocation, network robustness

## Abstract

With the development of information transmission, there is an increasing demand for state monitoring of fiber-optic communication networks to improve the security and self-healing ability of the network. Distributed optical fiber sensing is one of the most attractive methods because it can achieve real-time detection of the whole network without additional sensing heads. However, when the sensing network is introduced into the communication network, the failure probability should be efficiently suppressed with limited sensing resources. In this paper, the fuzzy logic system is used to evaluate the impact of different sensing resource allocation on optical cable network quality. The link failure probability and path failure probability under the condition of uniform and non-uniform sensing resource allocation are simulated and analyzed, respectively. As shown in the analysis results, the failure probability under non-uniform allocation is significantly lower than under uniform allocation. In this paper, we discussed and addressed the allocation of the optical fiber sensing and communication integrated (OFSCI) network with the limited sensing resource for the first time. The results are helpful to develop an allocation strategy for optical fiber sensing and a communication integrated network with a higher robustness.

## 1. Introduction

Optical fiber communication plays an important role in many fields, such as electrical power systems, environmental monitoring, infrastructure construction, etc. Therefore, the long-term security state of optical fiber networks is the basis to ensure the normal operation of the operation system. Detecting or predicting the security state of the whole network is an intuitive and efficient strategy. Distributed Optical Fiber Sensing (DOFS) is one of the most attractive methods, which can achieve physical state monitoring of the whole optical fiber network in real time. Besides, DOFS has the advantages of simple installation, low cost, high reliability [1,2,3,4,5,6,7,8,9,10], long-distance sensing ability [5,6,7,8], electromagnetic interference resistance [5,7], and multi-parameter measurement [1,4]. More importantly, DOFS can greatly reduce the difficulty of sensing terminal deployment and maintenance, and improve the security and stability of the optical fiber network. Therefore, many works have been proposed to integrate fiber-optic communication networks with DOFS technologies. The combination of a fiber-optic communication network and DOFS technologies is optical fiber sensing and the communication integrated (OFSCI) network. If a fiber-optic communication network can also act as a sensor network, OFSCI can be applied in many practical scenarios, such as power grids [11,12], operator fiber optic communication networks [13,14,15], the oil and gas industry [16,17], etc. In the scenarios mentioned above, fiber-optic communication network infrastructure can not only transmit communication signals but also various useful sensing data. This structure that integrated communication and sensing into the same optical fiber has brought higher social value. For example, OFSCI can realize the fault diagnosis of the communication network to improve the security of the network. It can also be used to achieve environmental monitoring that can serve smart cities and smart communities.

However, the OFSCI network is more sensitive to path failure than an ordinary fiber sensing network because in OFSCI the fiber sensing signal shares communication paths with other communication signals, instead of using its independent paths [14,18,19,20]. Thus, it is very important to theoretically assess each path’s failure probability in an OFSCI network and thereby reduce path failure probability before actually constructing the OFSCI. The problem is that the path failure probability in an OFSCI network may be affected by various factors, including link length, sensing data volume (i.e., sensing bandwidth requirement [21]), link order, and the number of sensing points [22]. The concept of this kind of complicated problem is fuzzy and does not have clear boundaries and definitions. There is a contradiction between precise mathematical language and vague habits of thought. It cannot be solved by a traditional mathematical model which resorts to precision of the problem. The fuzzy logic system can realize expert knowledge through fuzzy rules to make up for this defect. 

In this paper, we discussed and addressed the allocation of the optical fiber sensing and communication integrated (OFSCI) network with the limited sensing resource for the first time. We used the fuzzy logic system to assess failure probability of paths and links. The fuzzy logic system is an effective method to solve the contradiction between accuracy and validity [23]. Firstly, the principle of the fuzzy logic system is specifically demonstrated. Then, the link failure probability and path failure probability based on fuzzy logic system are simulated and analyzed, respectively. Moreover, the results of uniform allocation and non-uniform allocation of the limited sensing resources are compared. The experimental results show that the failure probability with non-uniform allocation is lower than that with the uniform allocation. In addition, the highest value of the simulation results of the lowest failure probability between any two nodes is decreased from 0.21 to 0.13 under non-uniform allocation, in the network consisting of 20 communication nodes. The method proposed in this paper can effectively analyze the failure probability of the OFSCI network with different sensing resource allocation. The results are helpful to develop the allocation strategy of the optical fiber sensing and communication integrated network with higher robustness.

## 2. Fuzzy Logic System

**Figure 1:**
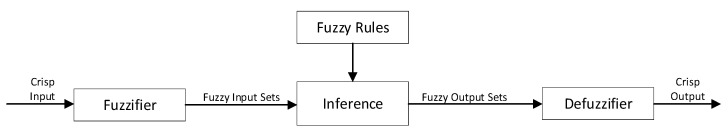
The schematic diagram of the fuzzy logic system.

The fuzzy logic system mainly includes four parts, which are fuzzifier, fuzzy rules, inference machine, and defuzzifier. The schematic diagram of the fuzzy logic system is shown in Figure 1. Firstly, the crisp input is transformed into fuzzifier and is set to the fuzzy input sets. Then, the fuzzy input sets are transformed into fuzzy output sets by fuzzy rules and inference. Finally, the fuzzy output sets are transformed into a crisp output by defuzzifier.

The fuzzifier is a mapping of setting real value points (crisp input) to fuzzy input sets by membership function. The value of membership function is between 0 and 1 on the closed interval, which is used to represent the corresponding degree of input variable to the fuzzy sets [24]. The fuzzy rules are the core of the fuzzy logic system and are composed by multiple IF-THEN conditional statements. The inference machine is used to transform the elements in the fuzzy input sets into the elements in the fuzzy output sets based on the fuzzy rules. The defuzzifier is a mapping from the fuzzy output sets to the real-valued points (crisp output) in the output sets. Considering computational complexity and implementation difficulty, we choose the mixed match scheme of single-valued fuzzification combined with product inference machine and center average defuzzification. 

Fuzzy logic systems have p inputs ($x_{1},\cdots ,x_{P}\in X$) and one output (y∈Y). $F_{1}^{},\cdots ,F_{p}^{}$ is the fuzzy input sets. $G^{}$ is the fuzzy output sets. Suppose there are M IF-THEN rules, the l-th(l=1,⋯,M) rule $R^{l}$ can be expressed as:

$R^{l}$: IF $x_{1}$ is $F_{1}^{l}$, $x_{2}$ is $F_{2}^{l}$, …, and $x_{p}$ is $F_{p}^{l}$, Then y is $G^{l}$.

Single-valued fuzzification refers to: Map the crisp input $X={\left(x_{1},\cdots ,x_{p}\right)}^{T}$ onto the fuzzy set $A_{X}={\left(x_{1}^{\prime },\cdots ,x_{p}^{\prime }\right)}^{T}$ in X. IF $x=x^{\prime }$, then $\mu_{A_{X}}\left(x\right)=1$, otherwise $\mu_{A_{X}}\left(x\right)=0$. $\mu_{A_{X}}\left(x\right)$ is the membership function of input. Generally, triangular or trapezoidal membership functions are used.

Each fuzzy rule represents a fuzzy implication relation. With product inference machine, assuming $F_{1}^{l}\times \cdots \times F_{p}^{l}=A^{l}$, then $R^{l}$ can be represented as

(1) $$R^{l}:F_{1}^{l}\times \cdots \times F_{p}^{l}\to G^{l}=A^{l}\to G^{l}\;\;l=1,\cdots ,M$$

where → represents mapping, × represents multiply. $R^{l}$ can be represented by the membership function $\mu_{R^{l}}\left(X,y\right)$.

(2) $$\mu_{R^{l}}\left(X,y\right)=\mu_{A^{l}\to G^{l}}\left(X,y\right)=\mu_{R^{l}}\left(x_{1},\cdots ,x_{p},y\right)=\mu_{F_{1}^{l}}\left(x_{1}\right)*\cdots *\mu_{F_{p}^{l}}\left(x_{p}\right)$$

The degree of firing level corresponding to the l-th rule is computed as:

(3) $$\mu_{F_{1}^{l}}\left(x_{1}\right)*\mu_{F_{2}^{l}}\left(x_{2}\right)*\cdots *\mu_{F_{p}^{l}}\left(x_{p}\right)=T_{i=1}^{p}\mu_{F_{i}^{l}}\left(x_{i}\right)$$

where ∗ and *T* both indicate the chosen t-norm. With center average defuzzification, crisp output is expressed as:

$$y(x)=\frac{\sum_{l=1}^{M}{\bar{y}}^{l}T_{i=1}^{p}\mu_{F_{i}^{l}}\left(x_{i}\right)}{\sum_{l=1}^{M}T_{i=1}^{p}\mu_{F_{i}^{l}}\left(x_{i}\right)}$$

where ${\bar{y}}^{l}$ is the center of the *l*-th output fuzzy set. 

The degree of each node is greater than or equal to 3 in the network topology, which consists of 20 communication nodes, 40 links. In order to find the transmission path with the lowest failure probability between any two communication nodes in the network, a fuzzy logic system can be utilized. Note that the failure probabilities are determined by sensing points, link lengths, link orders, and data volumes.

**Figure 2:**
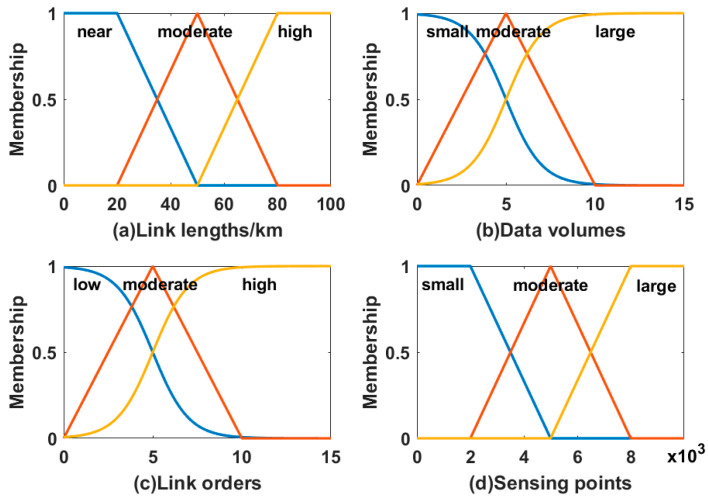
The fuzzy input sets mapping.

The four input variables (X) of fuzzy logic system are link lengths ($x_{1}$), link orders ($x_{2}$), data volumes ($x_{3}$), and sensing points ($x_{4}$), and the output variable (Y) is link failure probability (y). The input variables (X) are mapped to the fuzzy input sets ($F_{p}$) by the single value fuzzification method. The fuzzy input sets mapping relation of input variables is shown in Figure 2. Link lengths ($x_{1}$) have three fuzzy input sets of near ($F_{11}$), moderate ($F_{12}$), and high ($F_{13}$). Link orders ($x_{2}$) have three fuzzy input sets of low ($F_{21}$), moderate ($F_{22}$), and high ($F_{23}$). Data volumes ($x_{3}$) and sensing points ($x_{4}$) both have three fuzzy input sets of small ($F_{31}\&F_{41}$), moderate ($F_{32}\&F_{42}$), and large ($F_{33}\&F_{43}$).The fuzzy rules are the core of the fuzzy logic system, which consists of input variables and the output variable. The influence of input variables on output variables is shown in Table 1. There are 81 cases in the fuzzy rules as a group of the four input variables have three fuzzy input sets. By combining Equations (1)–(3), the fuzzy input sets ($F_{p}$) are transformed into fuzzy output sets (G). By equation (4), the fuzzy output sets (G) are transformed into crisp output (y) (i.e., link failure probability). The link failure probability calculates the path failure probability by using the dual method in probability theory.

## 3. Simulation and Analysis of Fuzzy Logic System

**Figure 3:**
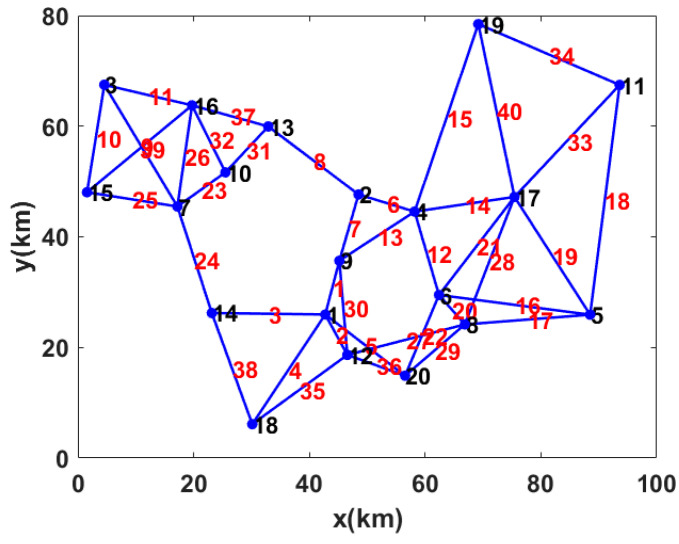
The basic topological structure of OFSCI network.

An OFSCI network model is established for simulation. For the communication part, it contains 20 communication nodes generated by Gaussian mixture distributed in a plane of 80 km × 100 km. In large-scale practical application scenarios, a fiber-optic communication network is usually a mesh structure [25]. The basic topological structure of the network is formed through link nodes, as shown in Figure 3. The black numeral represents the sequence number of the communication node, and the red numeral represents the link number which means the connection sequence of two communication nodes. The blue dots represent communication nodes, and the sensing points are distributed on the blue optical fiber links. For the sensing part, the number of sensing points is set as 200,000 to simulate the condition of limited sensing resources in OFSCI network. The uniform allocation and non-uniform allocation of the sensing points are applied, respectively, to simulate the limited sensing resources. Allocation rate refers to the sensing distance allocated to each sensing point. In uniform allocation mode, sensing resources are evenly allocated to links of different lengths, so the allocation rates on these links are different. In non-uniform allocation mode, sensing resources are allocated to links of different lengths according to the link length, so that the allocation rates of all links is the same. 

**Figure 4:**
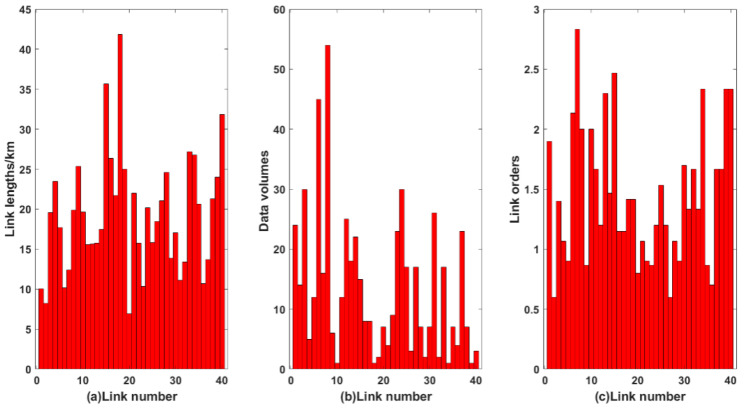
The relations of (**a**) link lengths, (**b**) data volumes, and (**c**) link orders with different link number.

The relations of independent variables (i.e., link lengths, data volumes and link orders with different link numbers) are shown in Figure 4. The link lengths in the OFSCI network model are calculated by the coordinate positions of communication nodes. The amount of link data volumes in the OFSCI network model is generated by Poisson allocation, and link orders in the OFSCI network model are calculated by Floyd’s shortest path algorithm. The distributions of the independent variables in Figure 4 are used to input into the fuzzy logic system, and the failure probability can be calculated.

### 3.1. Link Failure Probability in Different Allocation Modes

**Figure 5:**
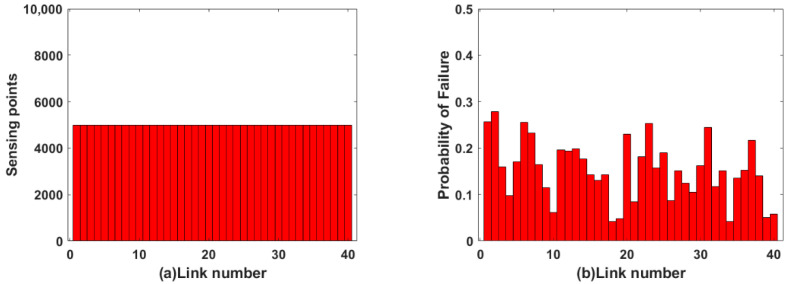
(**a**) The sensing points distribution in uniform allocation mode; (**b**) The distribution of link failure probability in uniform allocation mode.

For uniform allocation mode, 5000 sensing points are equidistant distributed on each link, as shown in Figure 5a. Because of different link lengths, the allocation rates of sensing points on different links is different. By inputting the data of four independent variables in Figure 4 and Figure 5a into the fuzzy logic system, the link failure probability of dependent variables in Figure 5b is obtained. The failure probability of each link of the OFSCI network output is demonstrated in Figure 5b. In Figure 5b, the vertical axis represents the failure probability of each link in the OFSCI network, which ranges from 0.1 to 0.3.

**Figure 6:**
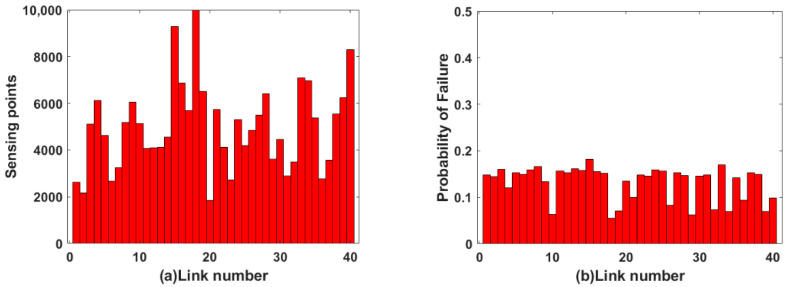
(**a**) The sensing points distribution in non-uniform allocation mode; (**b**) The distribution of link failure probability in non-uniform allocation mode.

For non-uniform allocation mode, the sensing points of each link are shown in Figure 6 to ensure the same sensing point allocation rates. By inputting the data of four independent variables in Figure 4 and Figure 6a into the fuzzy logic system, the link failure probability of dependent variables in Figure 6b is obtained. The failure probability of each link of the OFSCI network output is demonstrated in Figure 6b. In Figure 6b, the vertical axis represents the failure probability of each link in the OFSCI network, which ranges from 0.1 to 0.2.

The failure probability of each link under the two allocation modes are shown in Figure 5b and Figure 6b, respectively. As demonstrated in Figure 5b, 20% of links have a failure probability greater than 0.2. As shown in Figure 6b, the failure probability of all links is less than 0.2. It can be clearly observed that the failure probability of each link in the non-uniform allocation mode is lower than in the uniform allocation mode. This indicates that effective sensing resource allocation in an integrated fiber sensing and communication network helps to reduce link failure probability.

### 3.2. Path Failure Probability in Different Allocation Modes

For any two communication nodes, there is only one link, but there can be multiple paths through different links. In our simulation, the path failure probability is calculated by a fuzzy logic system combined with duality theory. In this subsection, a pair of nodes with sequence numbers 1 and 6 in Figure 3 is randomly selected as an example to calculate path failure probability under the uniform allocation mode and non-uniform allocation mode. Adjacency matrix A, if node *i* and node *j* is connected, $a_{ij}\;$= 1, otherwise it is 0. Starting from the start node *i*, first find a node *j* with $a_{ij}$ =1 and then continue to find the next node *k* with $a_{jk}$ = 1 from node *j*. Then, repeat this operation until the end node, and record the path. In this connected network structure, more than 7000 paths between node 1 and node 6 are obtained by traversing the adjacency matrix A. 

**Figure 7:**
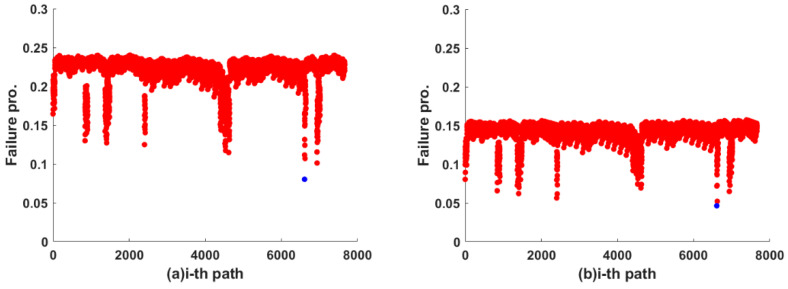
(**a**) Path failure probability between node 1 and node 6 in uniform allocation mode; (**b**) Path failure probability between node 1 and node 6 in non-uniform allocation mode.

According to the link failure probability output by the fuzzy logic system in two modes in Figure 5b and Figure 6b. the path failure probability between node 1 and node 6 can be simulated and calculated by traversing adjacency matrix and duality theory. The results are shown in Figure 7a,b. The blue dots in both Figures represent the path with the lowest failure probability. For uniform allocation mode, the failure probability of each path is mainly distributed between 0.1 and 0.25, and the lowest value is 0.08. For non-uniform allocation mode, the failure probability of each path is mainly distributed between 0.05 and 0.15, and the lowest value is 0.046. The results indicate that adjusting sensing resource allocation can reduce path failure probability to a certain extent, but the overall structure of the OFSCI network will not be changed.

**Figure 8:**
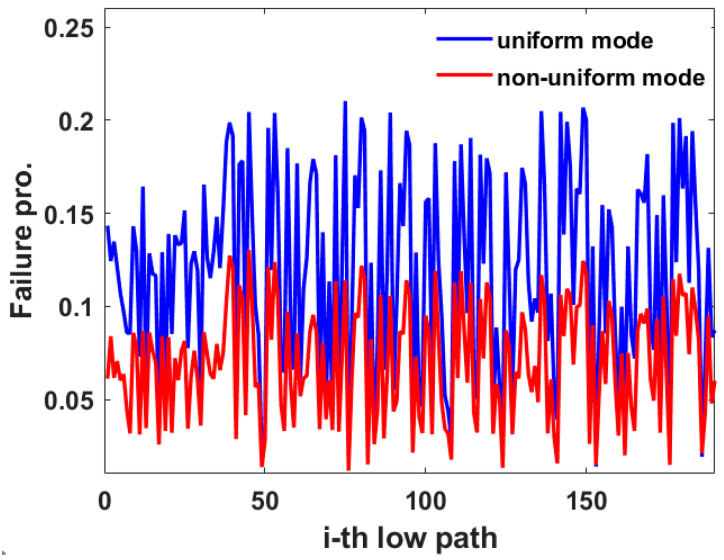
The simulation results of the lowest failure probability of any two nodes in the 20 communication nodes.

Furthermore, the path failure probability of 20 communication nodes is simulated, and the lowest failure probability of any 2 nodes. The results of the lowest failure probabilities are shown in Figure 8. As there are 20 communication nodes, the total number of paths with the lowest failure probability for 2 communication nodes is $C_{20}^{2}$(190). The blue curve is the result of the uniform mode. The red curve is the result of a non-uniform mode. It can be seen that the path failure probability of communication node pairs in non-uniform mode is smaller.

## 4. Conclusions

In this paper, the allocation of limited sensing resource in optical fiber sensing and in a communication integrated (OFSCI) network is discussed for the first time. We use the fuzzy logic system to assist sensing resource allocation for the OFSCI network. The link failure probability and path failure probability based on fuzzy logic system are simulated, respectively. And the results of uniform and non-uniform allocation with limited sensing resources are analyzed. The experimental results show that the failure probability with non-uniform allocation is lower than that with the uniform allocation. As a result, the highest value of the simulation results of the lowest failure probability between any two nodes is decreased from 0.21 to 0.13 under non-uniform allocation, in the network consists of 20 communication nodes. The method proposed in this paper can reasonably allocate the sensing resources and further improve the transmission robustness of the optical fiber sensing and communication integrated network. In the future, we may study the adaptive fuzzy logic system to realize the optimal sensing resource allocation for the OFSCI network. The adaptive fuzzy logic system is realized by combining a neural network and a fuzzy logic system. The neural network can make full use of model information and expert knowledge, which can effectively improve the automatic control ability of the fuzzy logic system.

## Figures and Tables

**Table 1:** Fuzzy rules.

Rules	Independent Variables				Dependent Variables
	Link Lengths	Link Orders	Data Volumes	Sensing Points	Degree of Failure
1	near	low	small	small	moderate
2	near	low	small	moderate	low
3	near	low	small	large	low
4	near	low	moderate	small	moderate
5	near	low	moderate	moderate	moderate
6	near	low	moderate	large	low
7	near	low	large	small	high
8	near	low	large	moderate	moderate
9	near	low	large	large	moderate
10	near	moderate	small	small	moderate
11	near	moderate	small	moderate	low
12	near	moderate	small	large	low
13	near	moderate	moderate	small	high
14	near	moderate	moderate	moderate	moderate
15	near	moderate	moderate	large	moderate
16	near	moderate	large	small	high
17	near	moderate	large	moderate	moderate
18	near	moderate	large	large	moderate
19	near	high	small	small	high
20	near	high	small	moderate	moderate
21	near	high	small	large	moderate
22	near	high	moderate	small	high
23	near	high	moderate	moderate	moderate
24	near	high	moderate	large	moderate
25	near	high	large	small	high
26	near	high	large	moderate	high
27	near	high	large	large	moderate
28	moderate	low	small	small	moderate
29	moderate	low	small	moderate	low
30	moderate	low	small	large	low
31	moderate	low	moderate	small	high
32	moderate	low	moderate	moderate	moderate
33	moderate	low	moderate	large	moderate
34	moderate	low	large	small	high
35	moderate	low	large	moderate	moderate
36	moderate	low	large	large	moderate
37	moderate	moderate	small	small	high
38	moderate	moderate	small	moderate	moderate
39	moderate	moderate	small	large	moderate
40	moderate	moderate	moderate	small	high
41	moderate	moderate	moderate	moderate	moderate
42	moderate	moderate	moderate	large	moderate
43	moderate	moderate	large	small	high
44	moderate	moderate	large	moderate	high
45	moderate	moderate	large	large	moderate
46	moderate	high	small	small	high
47	moderate	high	small	moderate	moderate
48	moderate	high	small	large	moderate
49	moderate	high	moderate	small	high
50	moderate	high	moderate	moderate	high
51	moderate	high	moderate	large	moderate
52	moderate	high	large	small	high
53	moderate	high	large	moderate	high
54	moderate	high	large	large	moderate
55	high	low	small	small	high
56	high	low	small	moderate	moderate
57	high	low	small	large	moderate
58	high	low	moderate	small	high
59	high	low	moderate	moderate	moderate
60	high	low	moderate	large	moderate
61	high	low	large	small	high
62	high	low	large	moderate	high
63	high	low	large	large	moderate
64	high	moderate	small	small	high
65	high	moderate	small	moderate	moderate
66	high	moderate	small	large	moderate
67	high	moderate	moderate	small	high
68	high	moderate	moderate	moderate	high
69	high	moderate	moderate	large	moderate
70	high	moderate	large	small	high
71	high	moderate	large	moderate	high
72	high	moderate	large	large	moderate
73	high	high	small	small	high
74	high	high	small	moderate	high
75	high	high	small	large	moderate
76	high	high	moderate	small	high
77	high	high	moderate	moderate	high
78	high	high	moderate	large	moderate
79	high	high	large	small	high
80	high	high	large	moderate	high
81	high	high	large	large	moderate

## Data Availability

Not applicable.
